# STARD13-correlated ceRNA network inhibits EMT and metastasis of breast cancer

**DOI:** 10.18632/oncotarget.8099

**Published:** 2016-03-15

**Authors:** Xiaoman Li, Lufeng Zheng, Feng Zhang, Jinhang Hu, Jinjiang Chou, Yu Liu, Yingying Xing, Tao Xi

**Affiliations:** ^1^ School of Life Science and Technology, China Pharmaceutical University, Nanjing, People's Republic of China; ^2^ Jiangsu Key Laboratory of Carcinogenesis and Intervention, China Pharmaceutical University, Nanjing, People's Republic of China; ^3^ Department of Biochemistry, School of Life Science and Technology, China Pharmaceutical University, Nanjing, People's Republic of China

**Keywords:** STARD13, 3’UTRs, ceRNA, breast cancer metastasis, EMT

## Abstract

Competing endogenous RNAs (ceRNAs) network has been correlated with the initiation and development of cancer. Here, we identify CDH5, HOXD1, and HOXD10 as putative STARD13 ceRNAs and they display concordant patterns with STARD13 in different metastatic potential breast cancer cell lines and tissues. Notably, 3’UTRs of these genes suppress breast cancer metastasis via inhibiting epithelial-mesenchymal transition (EMT) *in vitro* and *in vivo*, which are activated through the crosstalk between STARD13 and its ceRNAs in 3’UTR- and miRNA-dependent manners. In addition, Kaplan-Meier survival analysis reveals that mRNA level of STARD13 and its ceRNAs is remarkably associated with survival of breast cancer patients. These results suggest that 3’UTRs of CDH5, HOXD1, and HOXD10 inhibit breast cancer metastasis via serving as STARD13 ceRNAs.

## INTRODUCTION

Breast cancer is the most commonly cancer in women worldwide [1], As metastatic disease is responsible for as much as 90% of breast cancer-related mortality due to its surgically inoperable nature and the resistance of disseminated tumor cells to existing therapeutic agents [2, 3], it is vital to understand the basis for breast cancer metastasis. Recently, accumulating evidence indicates that miRNAs are closely linked to cancer metastasis by downregulating their targets post-transcriptionally [4–6]. However, in addition to being passive substrates for miRNA repression, the RNA targets themselves could decrease miRNA availability [7–9]. This reverse logic is extended to the notion that many miRNA targets act as ceRNAs to correlate with other targets through competing for miRNA binding [10, 11]. Experimental evidence for such a ceRNA crosstalk is initially described for the tumor-suppressor gene *PTEN*, which is increased by its pseudogene *PTENP1* in a miRNA-dependent manner [12]. Recent studies have revealed the potential physiological relevance of other ceRNAs, including the lncRNA HOTAIR or MEG3 that promotes or inhibits gastric cancer progression [13, 14], Hmga 3’UTR contributing to the transformation of lung cancer both *in vitro* and *in vivo* [15], as well as the circular RNAs (circRNAs) emerging as potent ceRNAs [16, 17]. Moreover, our laboratory has also investigated the roles of ceRNAs in cancer. Yang *et al.* [18] report that the FOXO1 3’UTR could increase E-cadherin levels by sequestering the shared miRNA miR-9, thus inhibiting breast cancer metastasis. Liu *et al*. [19] reveal that the AEG-1 3’UTR could induce EMT in human non-small cell lung cancer through its ceRNA activity. Zheng *et al*. [20, 21] report that the pseudogene CYP4Z2P 3’UTR exerts angiogenesis-promoting effect on breast cancer by acting as a ceRNA for CYP4Z1. And recently Zheng *et al*. [22] report that CXCR4 3’UTR functions as a ceRNA in promoting metastasis, proliferation and survival of MCF-7 cells by regulating miR-146a activity. In addition, our previous studies suggest that miR-125b could contribute to breast cancer metastasis *via* binding with STARD13 [23]. To elucidate the potential mechanisms by which the STARD13 3’UTR inhibits breast cancer metastasis, we have applied an integrated computational and experimental approach in the present study. Analyzing the miRNA binding sites located in the STARD13 3’UTR has identified numerous miRNAs binding with the STARD13 3’UTR that include the metastasis-promoting miRNAs: miR-9 [24, 25], miR-10b [26, 27] and miR-125b [23, 28]. We then seek to identify metastasis-related mRNAs that bind with the above three miRNAs with a high potential of translational repression. As a result, CDH5, HOXD1 and HOXD10 attract our interest. *CDH5* mutation is frequently found in metastatic triple-negative breast cancer [29]. *HOXD1* has been validated as a biomarker for diagnosis and prognosis of breast cancer *via* a functional hypermethylome screen [30], while HOXD10, as a direct target of miR-10b, suppresses cell migration and invasion in various types of cancer [31–34]. Though alterations in protein-coding genes govern cancer metastasis, the ceRNAs hypothesis challenges the idea that a protein-coding gene must be translated into a protein to exert its function and confers an additional non-protein-coding function to protein-coding mRNAs which underscores the function of the 3’UTRs [10, 35]. This promotes us to explore the functions of STARD13-, CDH5-, HOXD1-, and HOXD10-3’UTRs in breast cancer metastasis and whether they possess the functions through acting as ceRNAs.

Initially, we confirm the binding of miR-9, miR-10b, and miR-125b to STARD13 and the candidate ceRNAs, and validate the components of the ceRNA network. We next survey the correlation between the levels of the three common miRNAs and the STARD13 ceRNAs in breast cancer cells and tissues with distinct metastatic capabilities as well as the effect of STARD13- and its ceRNAs-3’UTRs on breast cancer metastasis *via* gain- and loss-of-function study *in vitro* and *in vivo*. Finally, we validate that STARD13 and its ceRNAs activate one another bidirectionally in a miRNA-dependent manner. To the best of our knowledge, the ceRNA network validated here hasn’t yet been reported anywhere. Therefore, STARD13- and its ceRNAs-3’UTRs might be used as combinatorial miRNA inhibitors for potential clinical applications, shedding fresh light on treatment of breast cancer.

## RESULTS

### Identification of STARD13 ceRNA candidates

**Figure 1:**
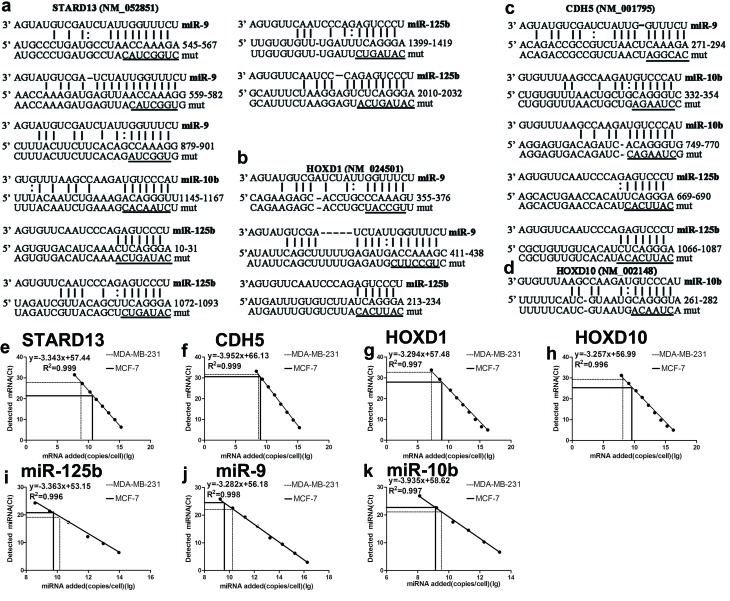
Prediction of STARD13 ceRNAs candidates and absolute miRNA and ceRNAs quantification in MCF-7 and MDA-MB-231 cells **a.** Computational analysis of STARD13 3’UTR showed potential binding sites for miR-9, miR-10b, and miR-125b. **b.** Computational analysis of HOXD1 3’UTR showed potential binding sites for miR-9 and miR-125b. **c.** Computational analysis of CDH5 3’UTR revealed potential binding sites for miR-9, miR-10b, and miR-125b. **d.** Computational analysis of HOXD10 3’UTR revealed potential binding sites for miR-10b. The mutated sequences in the seed regions of STARD13-, HOXD1-, CDH5-, and HOXD10-3’UTRs were underscored. **e.**-**k.** Absolute number of miRNAs and ceRNAs per cell were measured calibrated with an internal standard curve of synthetic miRNA or constructs containing ceRNAs-3’UTRs.

A computational approach was utilized to identify STARD13 ceRNAs in the human genome. Firstly, we sought to identify STARD13-binding miRNAs. Among the many STARD13-binding miRNAs, we focused on three validated breast cancer metastasis-promoting miRNAs: miR-9, miR-10b, and miR-125b. We then narrowed down our search to metastasis-related mRNAs that these miRNAs bind with a high potential of translational repression. As a result, we found CDH5-, HOXD1- and HOXD10-3’UTRs could bind to all or part of the three miRNAs (Figures 1a-1d). In addition, to assess the stoichiometric relationship between these three miRNAs and the predicted targets, we measured the absolute number of these entities per cell. qRT-PCR analysis combined with an internal standard curve revealed that miR-9, miR-10b, and miR-125b were expressed at 2.74×10^9^, 1.77×10^12^, 2.51×10^12^ molecules per cell in MCF-7 and 1.42×10^10^, 1.95×10^12^, 9.70×10^12^ per cell in MDA-MB-231 respectively, and STARD13, CDH5, HOXD1 and HOXD10 were expressed at 1.22×10^10^, 7.78×10^8^, 5.99×10^8^, 1.68×10^9^ molecules per cell in MCF-7 and 8.06×10^8^, 3.22×10^8^, 1.67×10^7^, 3.50×10^8^ per cell in MDA-MB-231 respectively (Figures 1e-1k). These three miRNAs had high miRNA: predicted targets ratios in both MCF-7 and MDA-MB-231 cells, which suggested miR-9, miR-10b and miR-125b binding efficacy tended to be high for STARD13, CDH5, HOXD1 and HOXD10 in these two cell lines.

### Confirmation of miRNAs binding with STARD13- and its ceRNAs-3’UTRs

**Figure 2:**
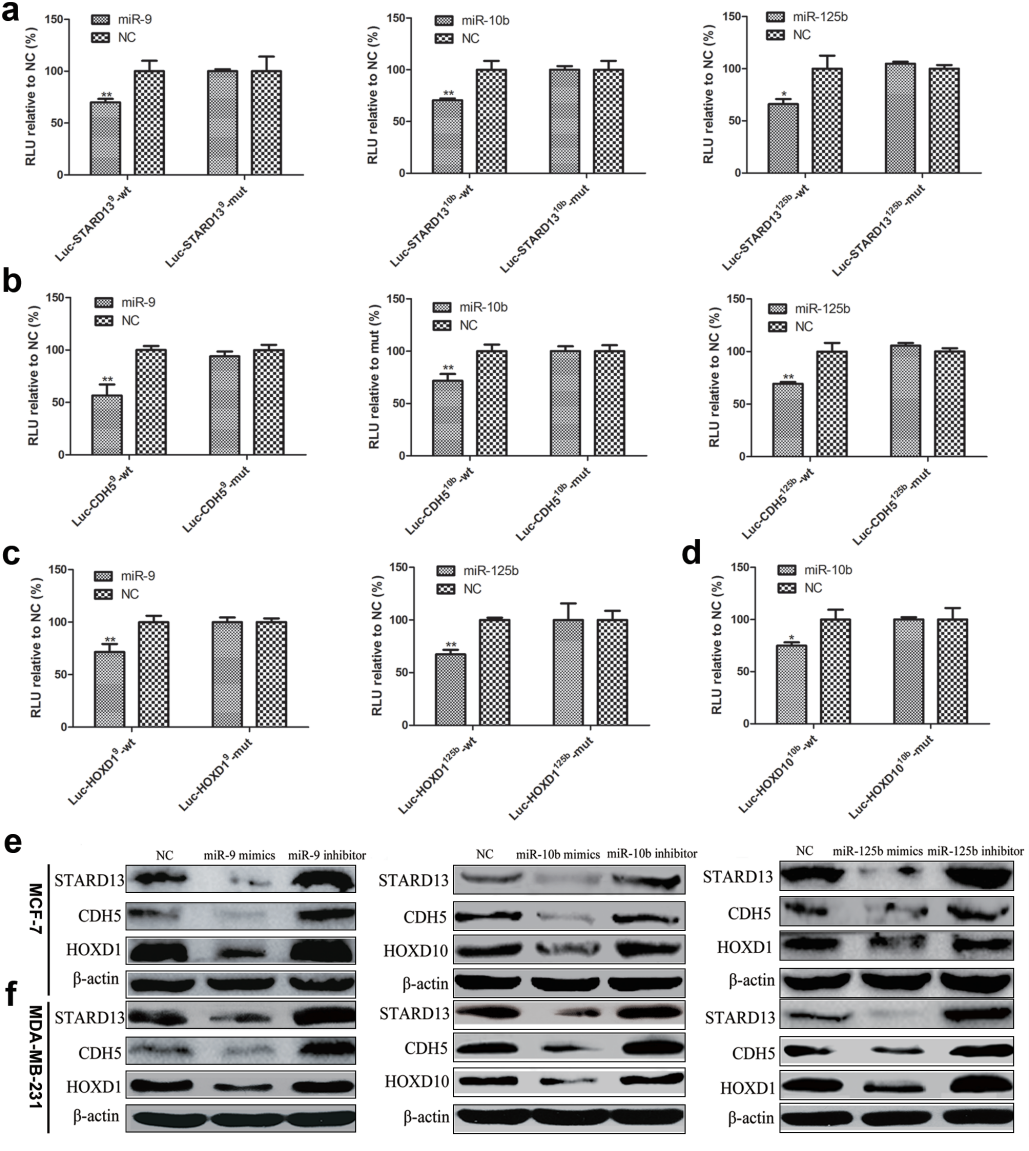
Confirmation of miRNAs binding with 3’UTRs of STARD13 and its ceRNAs **a.**-**d.** HEK293T cells were transfected with one of the luciferase report constructs of STARD13- **a.**, CDH5- **b.**, HOXD1- **c.**, and HOXD10-3’UTRs **d.** along with the corresponding miRNA or miRNA NC. In the presence of the targeting 3’UTR, luciferase activity was inhibited, which was reversed by the mutations of the potential miRNA target sites. Luciferase activity was normalized to β-gal. *n* = 3, *P < 0.05, **P < 0.01 *vs*. NC. **e.** Protein lysates prepared from MCF-7 cells transfected with miR-9 mimics/inhibitor, miR-10b mimics/inhibitor, and miR-125b mimics/inhibitor were subjected to western blot analysis. β-actin served as a loading control. **f.** Protein lysates prepared from MDA-MB-231 cells transfected with miR-9 mimics/inhibitor, miR-10b mimics/inhibitor, and miR-125b mimics/inhibitor were subjected to western blot analysis. β-actin served as a loading control.

The binding of miR-9, miR-10b and miR-125b to STARD13- and its ceRNAs-3’UTRs was detected by luciferase assays. For this purpose, HEK293T cells were co-transfected with one of the luciferase constructs containing 59bp fragments of the STARD13- and its ceRNAs-3’UTRs harboring wild-type (wt) or mutant binding sites (mut) for miR-9, miR-10b, and miR-125b and the corresponding miRNA or a negative control (NC), followed by luciferase activity analysis. As shown in Figure 2a-2d, luciferase activity was significantly repressed in the constructs of the STARD13- and its ceRNAs-3’UTRs when co-transfected with the corresponding miRNAs, as compared with NC. Mutation of the binding sites significantly reversed the repression. In addition, we constructed chimeric luciferase constructs tagged with the respective ceRNA-3’UTR fragments (Luc-ceRNA-3’UTR) harboring binding sites for all predicted miRNAs simultaneously (Supplementary Figure S1a). CDH1 and MAD1, which had been confirmed as the targets of miR-9 and miR-125b separately [18, 25, 36–38], were included as a positive control for miR-9 and miR-125b. And HOXD10 had been proved as the strong target of miR-10b in breast cancer and other cancers [32, 39, 40], which is consistent with our results, therefore the HOXD10 is also included as a positive control for miR-10b. As shown in Supplementary Figures S1b-S1d, a robust reduction of luciferase activity was observed in the Luc-ceRNA-3’UTR-expressing cells when co-transfected with the corresponding miRNAs, when compared with NC.

Furthermore, the ability of these miRNAs to suppress the expression of endogenous STARD13 and its ceRNAs was examined using miRNA mimics and inhibitors. The efficiency of upregulation and depletion of these miRNAs was examined by qRT-PCR analysis (Supplementary Figure S2). Overexpression of these miRNAs did not decrease the transcript levels of STARD13 and its ceRNAs in MCF-7 and MDA-MB-231 cells (Supplementary Figure S3); however, it did result in a significant reduction in protein levels of STARD13 and its ceRNAs (Figures 2e and 2f, compare lane 2 *versus* 1). Conversely, depletion of these miRNAs led to a modest increase in protein levels of STARD13 and its ceRNAs (Figures 2e and 2f, compare lane 3 *versus* 1) without affecting their transcript levels (Supplementary Figure S3). Altogether, these data demonstrated that these three miRNAs could inhibit the expression of STARD13 and its ceRNAs posttranscriptionally.

### The effects of STARD13 and its ceRNAs on breast cancer metastasis *in vitro*

**Figure 3:**
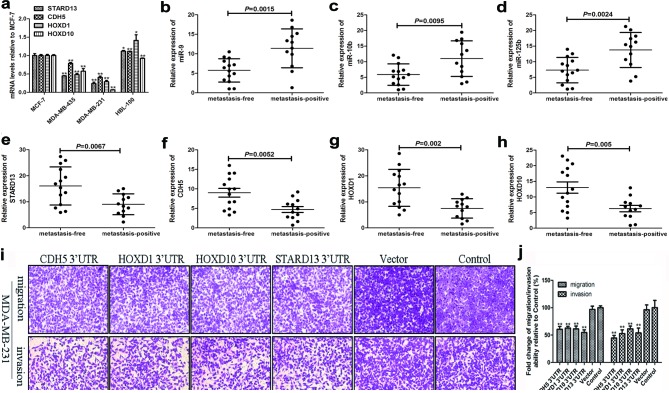
The effects of STARD13 and its ceRNAs on breast cancer metastasis *in vitro* **a.** Relative expression levels of STARD13, CDH5, HOXD1 and HOXD10 were examined by qRT-PCR normalized to GAPDH in four differently metastatic breast cancer cell lines. Data were presented as mean ± s.d.; *P < 0.05, **P < 0.01 *vs*. MCF-7. **b.**-**d.** Relative expression of miR-9 **b.**, miR-10b **c.**, and miR-125b **d.** was examined by qRT-PCR normalized to U6 in primary breast cancer with or without lymph node metastasis. **e.**-**h.** Relative expression levels of STARD13 **e.**, CDH5 **f.**, HOXD1 **g.** and HOXD10 **h.** were examined by qRT-PCR normalized to GAPDH in primary breast cancer with or without lymph node metastasis. **i.** Ectopic expression of 3’UTRs of STARD13, CDH5, HOXD1, and HOXD10 inhibited cell migration and invasion in MDA-MB-231 cells compared to the untreated control cells. **j.** Quantitation of cells migration and invasion shown in **i.**. Data were presented as mean ± s.d.; *P < 0.05, **P < 0.01 *vs*. Control.

It's reported that transcripts within a ceRNA network are co-regulated [41], we then tested whether STARD13 ceRNAs exhibited concordant expression patterns with STARD13 in breast cancer cell lines and tissues with different metastatic potential. As shown in Figure 3a, just like STARD13, mRNA levels of CDH5, HOXD1, and HOXD10 in MCF-7 cells (low metastatic) were 2.4-, 3.4-, and 14.8-fold higher than in MDA-MB-231 cells (high metastatic), respectively. Furthermore, levels of STARD13 and its ceRNAs were markedly lower in primary breast tumors with lymph node metastasis than those in the metastasis-free primary tumors (Figures 3e-3h), while the levels of miR-9, miR-10b, and miR-125b were significantly increased in metastatic tumors (Figures 3b-3d), suggesting that STARD13 and its ceRNAs might play a metastasis-suppressive role in breast cancer.

Based on the above observations, we speculated that STARD13- and its ceRNAs-3’UTRs could impair migration and invasion of breast cancer cells. We examined this effect by gain- and loss-of-function analyses. qRT-PCR and western blot analyses confirmed the efficient overexpression and knockdown of STARD13- and its ceRNAs-3’UTRs (Supplementary Figure S4a-S4c). Next, a series of functional assays were performed. As shown in Figures 3i and 3j, overexpression of STARD13- and its ceRNAs-3’UTRs significantly reduced cell migration and invasion compared to the untransfected control group. Further, the wound healing assay confirmed the inhibitory effect of STARD13- and its ceRNAs-3’UTRs on cell migration (Supplementary Figures S4d and S4e). In contrast, small interfering RNA (siRNA)-activated depletion of STARD13 (siSTARD13), CDH5 (siCDH5), HOXD1 (siHOXD1), and HOXD10 (siHOXD10) remarkably enhanced cell migration, invasion (Supplementary Figures S4f and S4g, and Figures S5a and S5b). Adhesion of tumor cells to extra-cellular matrix and basement membranes were considered to be the initial step in the invasive process for metastatic tumor cells [23]. As shown in Figures S5c, cells transfected with STARD13- and its ceRNAs-3’UTRs dramatically decreased the adhesive ability. Transfection with siSTARD13, siCDH5, siHOXD1, and siHOXD10 remarkably enhanced cell adhesion (Figures S5d). Moreover, live cell station also revealed that overexpression of STARD13- and its ceRNAs-3’UTRs significantly inhibited the migration of MDA-MB-231 cells (Supplementary Video 1–5). Collectively, these observations demonstrated that STARD13- and its ceRNAs-3’UTRs inhibited breast cancer metastasis *in vitro*.

### STARD13 and its ceRNAs suppressed breast cancer metastasis by inhibiting EMT

**Figure 4:**
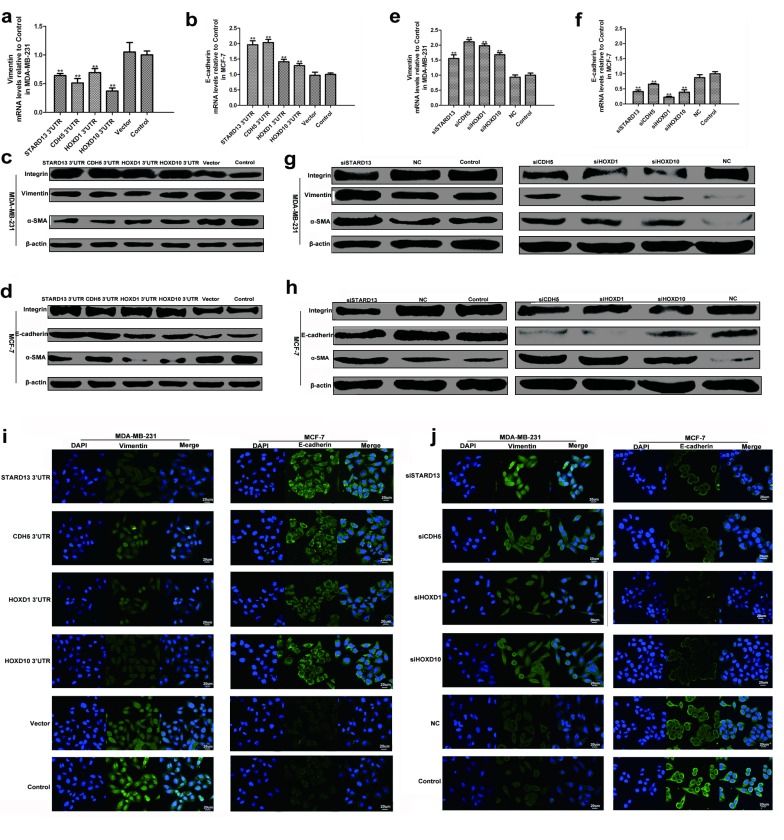
Effects of STARD13- and its ceRNAs-3’UTRs on EMT process **a.** and **b.** Ectopic expression of 3’UTRs of STARD13, CDH5, HOXD1, and HOXD10 decreased vimentin mRNA levels **a.** and increased E-cadherin mRNA levels **b.** measured by qRT-PCR normalized to GAPDH. **c.** and **d.** Ectopic expression of 3’UTRs of STARD13, CDH5, HOXD1, and HOXD10 decreased vimentin protein levels **c.** and increased E-cadherin protein levels **d.** analyzed by western blot relative to β-actin. **e.** and **f.** Knockdown of STARD13, CDH5, HOXD1, and HOXD10 increased vimentin mRNA levels **e.** and decreased E-cadherin mRNA levels **f.** measured by qRT-PCR normalized to GAPDH. **g.** and **h.** Knockdown of STARD13, CDH5, HOXD1, and HOXD10 increased vimentin protein levels **g.** and decreased E-cadherin protein levels **h.** analyzed by western blot relative to β-actin. **i.** MDA-MB-231 (left panel) and MCF-7 cells (right panel) treated with 3’UTRs of STARD13, CDH5, HOXD1, and HOXD10 expressed lower levels of vimentin and higher levels of E-cadherin compared to the untreated control group visualized by fluorescence microscopy. **j.** Knockdown of STARD13, CDH5, HOXD1, and HOXD10 elevated vimentin expression (left panel) and decreased E-cadherin expression (right panel) compared to the control group visualized by fluorescence microscopy. Green, vimentin was immunostained with anti-vimentin; blue, nuclei were stained with DAPI. **a.** and **b.**, **e.** and **f.** Data were presented as mean ± s.d.; **P < 0.01 *vs*. Control.

As miR-9, miR-10b, and miR-125b are confirmed to promoting breast cancer metastasis *via* enhancing EMT, we further tested whether STARD13- and its ceRNAs-3’UTRs exerted the metastasis-inhibitory effects through suppressing EMT. As shown in Figures 4a and 4b, STARD13- and its ceRNAs-3’UTRs transfected cells elevated mRNA level of epithelial marker E-cadherin and reduced mRNA level of mesenchymal marker vimentin. Accordingly, protein levels of E-cadherin and β-integrin were dramatically increased while vimentin and α-SMA protein levels were diminished (Figures 4c and 4d). Conversely, cells treated with siSTARD13, siCDH5, siHOXD1, and siHOXD10 induced EMT, as characterized by a decline in the expression of E-cadherin, β-integrin in tandem with an induction in the expression of vimentin and α-SMA (Figures 4e-4h). Immunofluorescent assay also showed that ectopic expression of STARD13- and its ceRNAs-3’UTRs decreased the expression of vimentin and increased the expression of E-cadherin, while knockdown of STARD13 and its ceRNAs elevated the expression of vimentin and reduced the expression of E-cadherin (Figures 4i and 4j). Altogether, these observations indicated that STARD13- and its ceRNAs-3’UTRs restrained breast cancer metastasis by inhibiting EMT process.

### 3’UTRs and miRNA dependency of reciprocal interaction of ceRNAs

**Figure 5:**
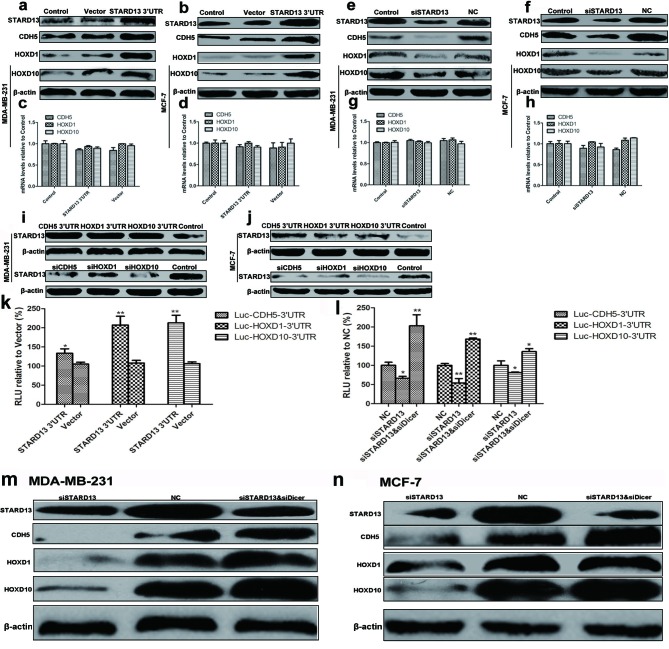
3’UTRs and miRNA dependency of reciprocal interaction of ceRNAs **a.** and **b.** Western blot analysis indicated that compared to the untreated control group, ectopic expression of STARD13 3’UTR upregulated protein levels of STARD13, CDH5, HOXD1, and HOXD10 in both MDA-MB-231 **a.** and MCF-7 cells **b.**. **c.** and **d.** qRT-PCR analysis indicated that compared to the untreated control group, ectopic expression of STARD13 3’UTR didn’t affect mRNA levels of CDH5, HOXD1, and HOXD10 in both MDA-MB-231 **c.** and MCF-7 cells **d.**. **e.** and **f.** Western blot analysis revealed that protein levels of STARD13, CDH5, HOXD1, and HOXD10 decreased as a result of transfection with siSTARD13. **g.** and **h.** qRT-PCR analysis indicated that compared to the untreated control group, knockdown of STARD13 3’UTR didn’t affect mRNA levels of CDH5, HOXD1, and HOXD10 in both MDA-MB-231 **g.** and MCF-7 cells **h.**. **i.** and **j.** Protein level of STARD13 increased as a result of ectopic expression of its ceRNAs 3’UTRs (upper panel), while knockdown of its ceRNAs decreased the expression of STARD13 (lower panel) in both MDA-MB-231 **i.** and MCF-7 cells **j.**. **k.** Luciferase activity in MCF-7 cells co-transfected with STARD13 3’UTR and a Luc-ceRNA-3’UTR reporter construct. Ectopic expression of STARD13 3’UTR in Luc-ceRNA-3’UTRs-expressing cells increased luciferase activity compared to the control vector group. **l.** Luciferase activity in MCF-7 cells co-transfected with siSTARD13 and a Luc-ceRNA-3’UTR reporter construct. siSTARD13-activated knockdown of STARD13 in Luc-ceRNA-3’UTR-expressing cells decreased luciferase activity compared with siRNA NC. However, in the presence of siDicer, the effects were reversed. Data were presented as mean ± s.d.; *P < 0.05, **P < 0.01 *vs*. Control. (m and n) MDA-MB-231 **m.** and MCF-7 cells **n.** were transfected with siSTARD13 or along with siDicer followed by western blot assays. Although knockdown of STARD13 decreased protein levels of CDH5, HOXD1, and HOXD10, siDicer reversed the effects. However, despite in the presence of siDicer, siSTARD13 still reduced protein level of STARD13.

We hypothesized that an increase in STARD13 3’UTR level would bind to and arrest the functions of these three miRNAs, followed by an increased translation of STARD13 and its ceRNAs. As shown in Figures 5a-5d, ectopic expression of STARD13 3’UTR did result in a significant upregulation of the protein levels of STARD13 and its ceRNAs without affecting their transcript levels. Conversely, siSTARD13 led to a marked reduction in expression of STARD13 ceRNAs at protein level but not mRNA level (Figures 5e-5h). These results suggested that STARD13 3’UTR increased the expression of the STARD13 ceRNAs at translational level rather than transcriptional level. Next, the ability of STARD13 ceRNAs-3’UTRs to promote STARD13 expression was investigated. As indicated in Figures 5i and 5j, overexpression of STARD13 ceRNAs-3’UTRs increased STARD13 protein levels, and knockdown of STARD13 ceRNAs significantly reduced STARD13 protein levels, thus identifying the regulatory loops between STARD13 and its ceRNAs.

To ascertain whether the protein levels of STARD13 ceRNAs was stimulated *via* their 3’UTRs, MCF-7 cells were co-transfected with Luc-ceRNA-3’UTR as well as STARD13 3’UTR or siSTARD13. As indicated in Figure 5k and 5l, overexpression of STARD13 3’UTR significantly elevated luciferase activity in Luc-ceRNAs-3’UTRs co-transfected cells. Conversely, knockdown of STARD13 markedly lowered luciferase activity in the cells. Moreover, ectopic expression of STARD13-coding region (CDS) increased STARD13 protein level, but did not affect protein levels of STARD13 ceRNAs (Supplementary Figure S6a. These results suggested that STARD13 increased the protein levels of its ceRNAs through their 3’UTRs.

To investigate the miRNA dependency of STARD13-correlated ceRNA regulation, we utilized siRNA (siDicer) to downregulate Dicer1. As Dicer1 is a critical enzyme involved in the processing of mature miRNAs, knockdown of Dicer1 presents an ideal system to evaluate miRNA-dependent effects. qRT-PCR analysis revealed the efficient knockdown of Dicer1 and the concomitant downregulation of miR-9, miR-10b, and miR-125b (Supplementary Figure S6b and S6c). Notably, downregulation of protein levels of STARD13 ceRNAs by STARD13 knockdown was profoundly attenuated and even reversed in siDicer-treated cells compared with NC (Figures 5m and 5n). Similarly, in the presence of siDicer, the decreased luciferase activity of Luc-ceRNA-3’UTRs transfected cells inhibited by siSTARD13 was reversed (Figure 5l), suggesting that mature miRNAs were essential for STARD13-correlated ceRNAs regulation.

### STARD13 and its ceRNAs suppressed metastasis of breast cancer in a xenograft model

**Figure 6:**
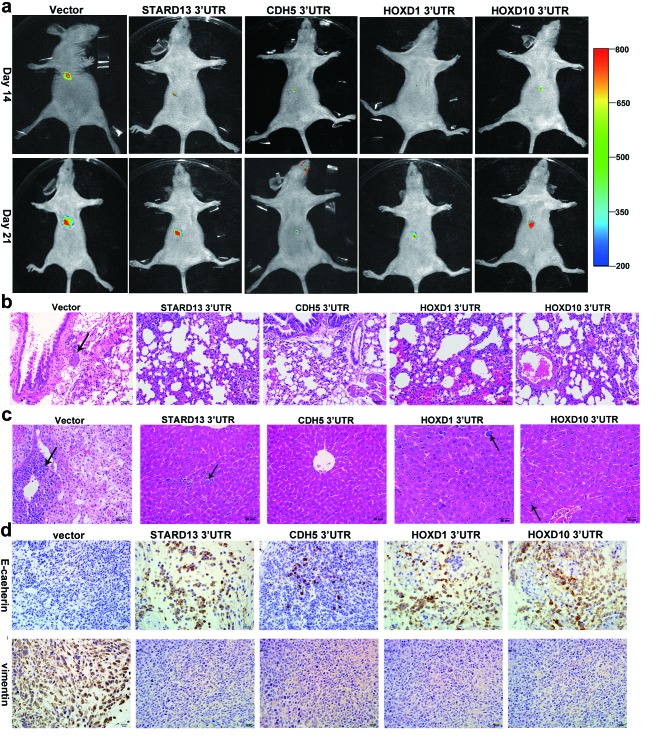
Effects of STARD13- and its ceRNAs-3’UTRs on metastasis and EMT of breast cancer in a xenograft model **a.** Whole animal imaging analysis of mice (*n* = 5 per group) injected with MDA-MB-231 cells stably overexpressing STARD13-, CDH5-, HOXD1-, and HOXD10-3’UTRs. Ventral views were shown for all five groups on day 14 and day 21. **b.** and **c.** HE-staining of lungs **b.** and livers **c.** isolated from mice that received tail vein injection of MDA-MB-231 cells stably overexpressing STARD13-, CDH5-, HOXD1-, and HOXD10-3’UTRs (*n* = 5 per group). Magnification ×200. Scale bars 50 μm. **d.** Immunohistochemical staining of E-cadherin (upper panel) and vimentin (lower pannel) in tumors subcutaneously injected with MDA-MB-231 cells stably transfected with STARD13-, CDH5-, HOXD1-, and HOXD10-3’UTRs or an empty vector. The brown signal corresponded to E-cadherin or vimentin. Magnification ×400. Scale bars 20 μm.

To explore whether overexpression of STARD13- and its ceRNAs-3’UTRs could also inhibit breast cancer metastasis *in vivo*, a nude mice xenograft model was applied using MDA-MB-231 cells stably transfected with STARD13- and its ceRNAs-3’UTRs or an empty vector. qRT-PCR analysis demonstrated the efficient overexpression of STARD13- and its ceRNAs-3’UTRs (Supplementary Figure S7a). The stably transfected cells were transplanted into BALB/c nude mice *via* tail vein injection, and the carestream (Carestream Health Inc., Toronto, Canada) noninvasive optical imaging system was used for whole animal imaging. The mice injected with STARD13- and its ceRNAs-3’UTRs had fewer metastasis signals in ventral views than the mice injected with empty vector cells (Figure 6a). Then the mice were killed, and the lungs and livers were collected and subjected to hematoxylin and eosin (HE)-staining analysis to evaluate tissue morphology. HE-staining elucidated fewer pulmonary and hepatic metastatic nodules in STARD13- and its ceRNAs-3’UTRs groups than those in the empty vector group (Figures 6b and 6c), suggesting a metastasis-suppressive role of STARD13- and its ceRNAs-3’UTRs *in vivo*. In addition, the stably transfected cells were injected subcutaneously into each flank of nude mice, and all mice were killed 2 weeks later to harvest the xenografts for analysis of EMT markers. As shown in Figure 6d, expression level of E-cadherin was higher in STARD13- and its ceRNAs-3’UTRs tumors than that in control tumors, whereas vimentin expression level was decreased in STARD13- and its ceRNAs-3’UTRs tumors. We further tested whether the reciprocal ceRNAs interaction occurred *in vivo* as well. Expectedly, the protein levels of STARD13 and its ceRNAs were remarkably upregulated in the tumors derived from the STARD13 3’UTR-overexpressing cells (Supplementary Figure S7b), and overexpression of the STARD13 ceRNAs-3’UTRs could elevate STARD13 protein level (Supplementary Figure S7c).

### The association between mRNA levels of STARD13 ceRNAs and the survival of breast cancer patients

**Figure 7:**
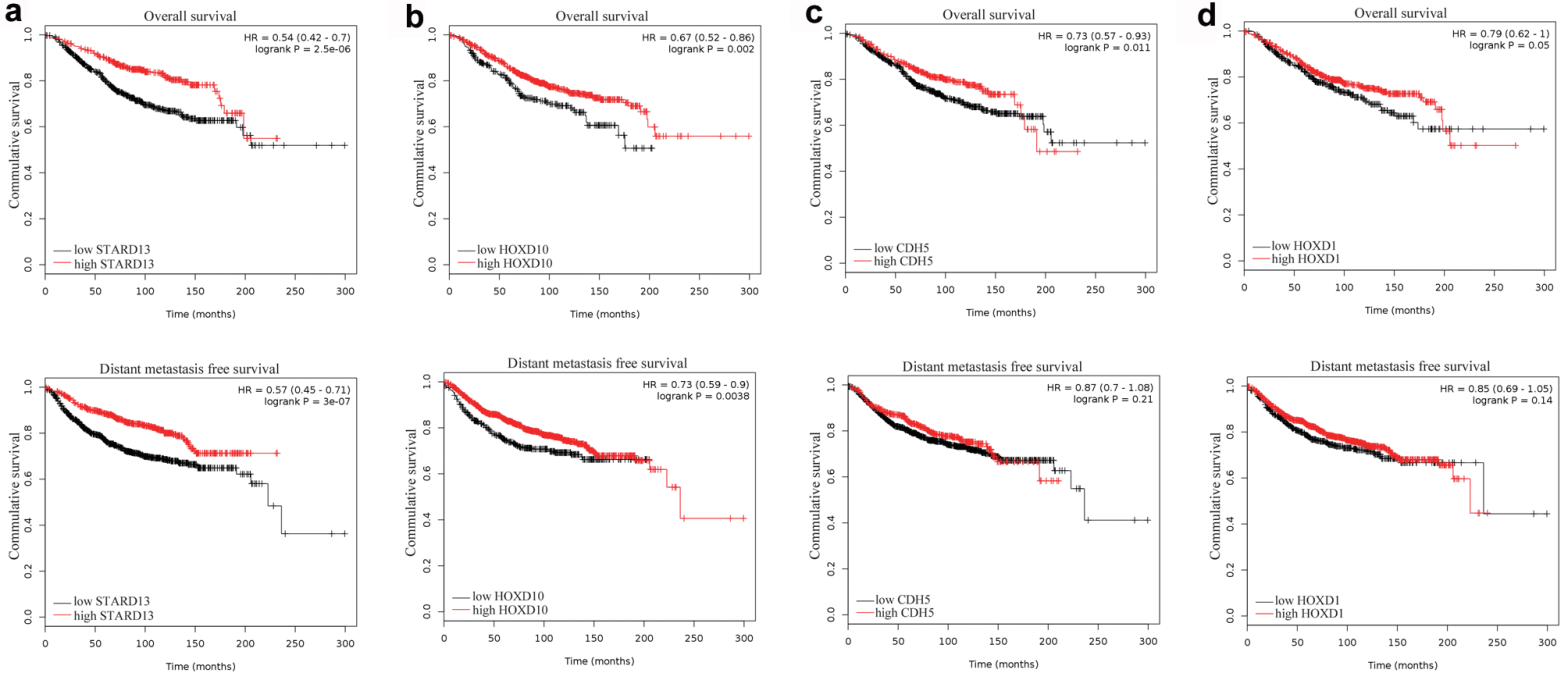
Correlation between the levels of STARD13 ceRNAs and the survival of breast cancer patients **a.**-**d.** KM survival curves based on analysis of a published microarray dataset from breast cancer patients showed OS and DMFS of patients separated into low and high STARD13 **a.**, HOXD10 **b.**, CDH5 **c.** and HOXD1 **d.** expression.

The KM plotter tool was employed to assess if mRNA levels of STARD13 and its ceRNAs correlated with the survival of breast cancer patients. As shown in Figure 7a and 7b, high expression of STARD13 and HOXD10 was significantly correlated with longer overall survival (OS) (log-rank *p* = 0 and 0.002, respectively) and distant metastasis-free survival (DMFS) (log-rank *p* = 0 and 0.004, respectively), which expands our *in vitro* and *in vivo* observations described above. High expression of CDH5 was significantly associated with increased OS only (log-rank *p* = 0.011, Figure 7c, upper panel), and no significant survival difference was detected between the high- and low-HOXD1 expressing groups (Figure 7d). Overall, these data indicated that mRNA levels of STARD13 and its ceRNAs were remarkably correlated with the survival of breast cancer patients.

## DISCUSSION

Increasing experimental evidence discloses that ceRNA activity is correlated with the development of cancers [42–44]. Our previous study uncovers that miR-125b could promote breast cancer metastasis by binding with STARD13 *in vitro* and *in vivo*, prompting us to explore whether some other transcripts may serve as STARD13 ceRNAs to coordinately suppress breast cancer metastasis.

Initially, STARD13-binding miRNAs are successfully identified. We focus on miR-9, miR-10b, and miR-125b since their “total context+scores” calculated by TargetScan are all below −0.2, indicating that they could bind with the STARD13 3’UTR relatively strong. Importantly, previous studies establish that miR-9 [25], miR-10b [40] and miR-125b [23] could drive the metastatic process of breast cancer. Next, STARD13, CDH5, HOXD1, and HOXD10 are identified as direct targets of these miRNAs by employing luciferase assay, qRT-PCR and western blot. Unexpectedly, the mRNA levels of STARD13, CDH5, HOXD1, and HOXD10 show marginal changes following upregulation or downregulation of miR-9, miR-10b and miR-125b (Figure S3), probably due to the effects of miRNAs on mRNA could change from preventing translation to affecting mRNA half-life [45]. Alternatively, miR-9, miR-10b and miR-125b could just inhibit the translation of STARD13, CDH5, HOXD1, and HOXD10 mRNA. Meanwhile, protein levels of STARD13, CDH5, HOXD1, and HOXD10 display little difference following knockdown of miR-9, miR-10b and miR-125b (Figures 2e and 2f, compare lane 3 *versus* 1), probably due to the other endogenous miRNAs could also bind with these mRNAs and thereby repressed their translation. Our studies further show that STARD13 ceRNAs display concordant expression patterns with STARD13 in differently metastatic breast cancer cells (Figure 3a), supporting that they all have strong sponging effects and intense reciprocal ceRNAs interaction (Figure 5) [46]. It is well documented that EMT aberrantly occurs during tumor progression, which allows cancer cells to lose their epithelial cell-like characteristics and to alternatively adopt a mesenchymal or fibroblast-like phenotype, thus initiates the invasion and metastasis cascade [47, 48]. In the present study, we have discovered that ectopic expression of STARD13- and its ceRNAs-3’UTRs strongly suppresses breast cancer metastasis *in vitro via* inhibiting EMT (Figures 3 and 4). These results validate that the effect of STARD13- and its ceRNAs-3’UTRs on cancer metastasis is activated through the bidirectional crosstalk between STARD13 and its ceRNAs, an important corollary of the ceRNA interaction [10]. We also apply a xenograft model to verify the inhibitory roles that STARD13- and its ceRNAs-3’UTRs play *in vivo* (Figures 6a-6c). In sum, these data testify our hypothesis that STARD13, CDH5, HOXD1, and HOXD10 may bind with miR-9, miR-10b and miR-125b to suppress breast cancer metastasis.

With the presentation of ceRNA hypothesis, assigning functions to non-coding transcripts will, therefore, undoubtedly lead to important insight into basic physiology and disease progression [49–51]. Of particular interest, since some genes are relatively large in size and it's difficult to study their functions by traditional molecular cloning techniques, using non-coding transcripts which are relatively small in size to study gene functions could partially overcome the limitation. For instance, ectopic expression of the versican 3’UTR provides an ideal approach to study the functions of versican V1 isoform which was difficult to clone due to its large size [52]. Similarly, we previously use the CYP4Z2P 3’UTR as a model to explore the role of pseudogene CYP4Z2P in breast cancer angiogenesis as the coding sequences of CYP4Z2P was difficult to obtain [20].

Since our previous study confirm that the 3’UTR of transcription factor FOXO1 could repress the metastasis of breast cancer cells *via* regulating miR-9 activity, future work is required to explore whether a broader ceRNA network (ceRNET) involving FOXO1 and its targets exists to coordinate the STARD13 ceRNAs in inhibiting breast cancer metastasis. In addtition, due to E-cadherin is identified as a target of miR-9 by our group before and the levels of E-cadherin and vimentin are measured in this work, further supports should be needed to confirm that EMT regulation underlies the effects of STARD13 and its ceRNAs in cancer cells and to establish a direct link between this RNA network and EMT phenotypes.

Apparently, as STARD13 and its ceRNAs harbor numerous MREs for other different miRNAs, and miR-9, miR-10b and miR-125b typically repress other multiple target transcripts, the STARD13 ceRNAs likely act in complex networks. It is possible that thousands or hundreds of RNA components which includes mRNAs, transcribed pseudogenes, long noncoding RNAs (lncRNA), circular RNA (circRNA) may exist in this ceRNA pool and STADR13, CDH5, HOXD1 and HOXD10 might only represent a very small part of the ceRNETs. Despite multiple relationships, our study provide solid evidences to outline the novel roles of STARD13-, CDH5-, HOXD1-, and HOXD10-3’UTRs in modulating breast cancer metastasis *via* competing for miR-9, miR-10b and miR-125b *in vitro* and *in vivo*. These data suggest that targeting these three miRNAs may be a potential therapeutic approach for the treatment of breast cancer metastasis. Given the crucial role of STARD13-, CDH5-, HOXD1-, and HOXD10-3’UTRs in breast cancer metastasis, we believe that STARD13-, CDH5-, HOXD1-, and HOXD10-3’UTRs could be therapeutic targets for the development of anti-metastatic strategy for breast cancer treatment in the near future.

## MATERIALS AND METHODS

### Cell culture and patient samples

HEK293T cells and human breast cancer cells MCF-7, MDA-MB-231 were cultured in DMEM medium (Gibco, Grand Island, NY, USA) supplemented with 10% FBS (Gibco), 80 U/ml penicillin and 0.08 mg/ml streptomycin at 37°C under humidified atmosphere with 5% CO_2_. Cell line authentication was assessed using short tandem repeat (STR) DNA profiling method every year in our laboratory and the latest verification was done in June 2015. Twelve primary breast tumors with lymph node metastasis and fourteen metastasis-free primary breast tumors were obtained from Zhongda Hospital Southeast University from February 2014 to December 2014. Approval from the Institute Research Ethics Committee was obtained for the use of these clinical materials for research purposes.

### Transfection

For the transfection of siRNAs or miRNA mimics/inhibitors (Biomics Biotechnology Inc., China), cells that reached 50% confluence were transfected with synthetic siRNAs or miRNA mimics/inhibitors at a final concentration of 50 nM using Lipofectamine 2000 (Invitrogen, Carlsbad, CA) in 6-well plates according to the manufacturer's recommendations for transfection. And a universal negative control siRNA (siRNA NC) or a negative control mimics/inhibitor (miRNA NC) (Biomics Biotechnology Inc., China) was used. The sequences of the inhibitors are blow: miR-9 inhibitor: UCAUACAGCUAGAUAACCAAAGA; miR-10b inhibitor: CACAAAUU

CGGUUCUACAGGGUA; miR-125b inhibitor: UCACAAGUUAGGGUCUCAGGG

A. Detailed procedure was described elsewhere [18].

### Plasmid construction

The 3’UTRs of STARD13, CDH5, HOXD1, and HOXD10 were cloned into the XhoI and KpnI sites of the pcDNA3.1 or the HindIII and BamHI sites of the pSilencer4.1-CMV to upregulate the levels of the STARD13 ceRNAs-3’UTRs, refered as ceRNAs-3’UTR. The STARD13 ceRNAs-3’UTRs, CDH1 3’UTR and MAD1 3’UTR were then subcloned into a luciferase reporter vector (pMIR-Report, Ambion, Carlsbad, CA, USA) using SpeI and MluI or HindIII and SpeI restriction sites for luciferase assays, and the corresponding plasmids were denoted as Luc-ceRNA- 3’UTR, Luc-CDH1-3’UTR, Luc-MAD1-3’UTR. Primer sequences were described in Table S1.

### RNA extraction and qRT-PCR

Detailed procedures for RNA extraction and qRT-PCR for mRNA and miRNA analysis were described elsewhere [19]. Specific primers used in this experiment were described in Table S2.

### Wound healing assay

Detailed procedure was described elsewhere [18].

### Migration and invasion assays

The transwell migration and invasion assays were performed as described previously [19].

### Adhesion assay

Detailed procedure was described elsewhere [18].

### Live cell Imaging System

Scratch-based migration assays were carried out with an Olympus automatic system (Olympus Corporation, Tokyo, Japan) according to the manufacturer's protocol. Briefly, monolayers of MDA-MB-231 cells transfected with STARD13- and its ceRNAs-3’UTRs were scratched using a scratching apparatus that produced highly identical scratches in each well. The Olympus system was programmed to obtain real-time phase contrast images of the wounds at 24 time points, i.e., images were taken every 1 hour for 24 hours.

### Western blot analysis

Detailed procedure was described elsewhere [20].

### miRNA target prediction

The public databases TargetScan 6.2 (http://www.targetscan.org/) and RNA22 (http://www.mirbase.org/) were used to identify putative miRNA seed-matching sequences in the STARD13-, CDH5-, HOXD1- and HOXD10-3’UTRs.

### Luciferase reporter assay

For miRNA target validation assays, pMIR-Report vector was used to introduce the 59bp fragments of the STARD13-, CDH5-, HOXD1-, and HOXD10-3’UTRs containing the wild-type (wt) and mutant binding sites (mut) for miR-9, miR-10b, and miR-125b as described previously [23]. To investigate the 3’UTR dependency of STARD13-correlated regulation of ceRNAs, MCF-7 cells were transfected with 100 ng of pcDNA3.1-STARD13 3’UTR or the empty vector pcDNA3.1, and 500 ng of Luc-ceRNA-3’UTR. On the other hand, 50 nM siSTARD13 or siRNA NC were co-transfected with 200 ng of Luc-ceRNA-3’UTR. To explore the miRNA dependency of STARD13-correlated regulation of ceRNAs, 50 nM siDicer was co-transfected with siSTARD13 to knockdown miRNA and STARD13 at the same time. In all cases, β-gal was used as a normalization control for transfection efficiency. Luciferase activity was measured as described previously [18].

### Immunofluorescent assay

Detailed procedure was described elsewhere [23].

### *In vivo* metastasis and immunohistochemical assays

All animal experiments were performed with the approval of Ethics Committee for Animal Experimentation of China Pharmaceutical University. To produce experimental metastasis, MDA-MB-231 cells (1×10^6^) that were tagged with luciferase gene and that stably overexpressed STARD13-, CDH5-, HOXD1-, HOXD10-3’UTRs or empty vector were injected into the tail veins of 5-week-old BALB/c female nude mice (5 mice per group, total 25 mice). On day 14 and day 21, whole animal imaging was monitored after intraperitoneal D-Luciferin potassium salt injection (150 mg/kg bodyweight) using the carestream noninvasive optical imaging system. Then all the mice were killed. The lungs and livers were collected, fixed in 10% formalin for 48 h at room temperature and embedded in paraffin. HE-staining was performed on sections from embedded samples for tissue morphology evaluation. For xenograft tumor model studies, 3×10^6^ model cells were injected subcutaneously into each flank of 5-week-old BALB/c female mice (5 mice per group, total 25 mice). After 2 weeks, mice were killed and tumor tissues were collected for western blot analysis or fixed in 10% formalin for 48h at room temperature and embedded in paraffin for immunohistochemical assay.

### Clinical data

The KM Plotter tool (http://kmplot.com/analysis/), a meta-analysis-based biomarker assessment tool, was used to compare the survival of breast cancer patients whose STARD13, CDH5, HOXD1 and HOXD10 mRNA levels were in the top 1/3 (high) *vs*. the bottom 1/3 (low) groups in publicly available breast cancer gene expression data (Affymetrix (Santa Clara, CA, USA) ProbeID ) selected on the basis of the following parameters: overall survival (OS, 1115 patients) or distant metastasis-free survival (DMFS, 1609 patients), upper *versus* lower tertile of STARD13, CDH5, HOXD1, and HOXD10 expression.

### Statistical analysis

Results were reported as mean ± s.d. and statistically compared using unpaired Student's *t* test. Values of *P* < 0.05 were considered statistically significant. *P < 0.05, **P < 0.01.

## SUPPLEMENTARY MATERIAL FIGURES AND TABLES












