# Supplementary material for: STARD13-correlated ceRNA network inhibits EMT and metastasis of breast cancer
Source: Oncotarget. 2016 Mar 15;7(17):23197–211. doi: 10.18632/oncotarget.8099 (PMC5029620; doi:10.18632/oncotarget.8099)
Supplement: Supplementary file 1 [file oncotarget-07-23197-s001.pdf]

STARD13-correlated ceRNA network inhibits EMT and metastasis of breast cancer

Supplementary Material

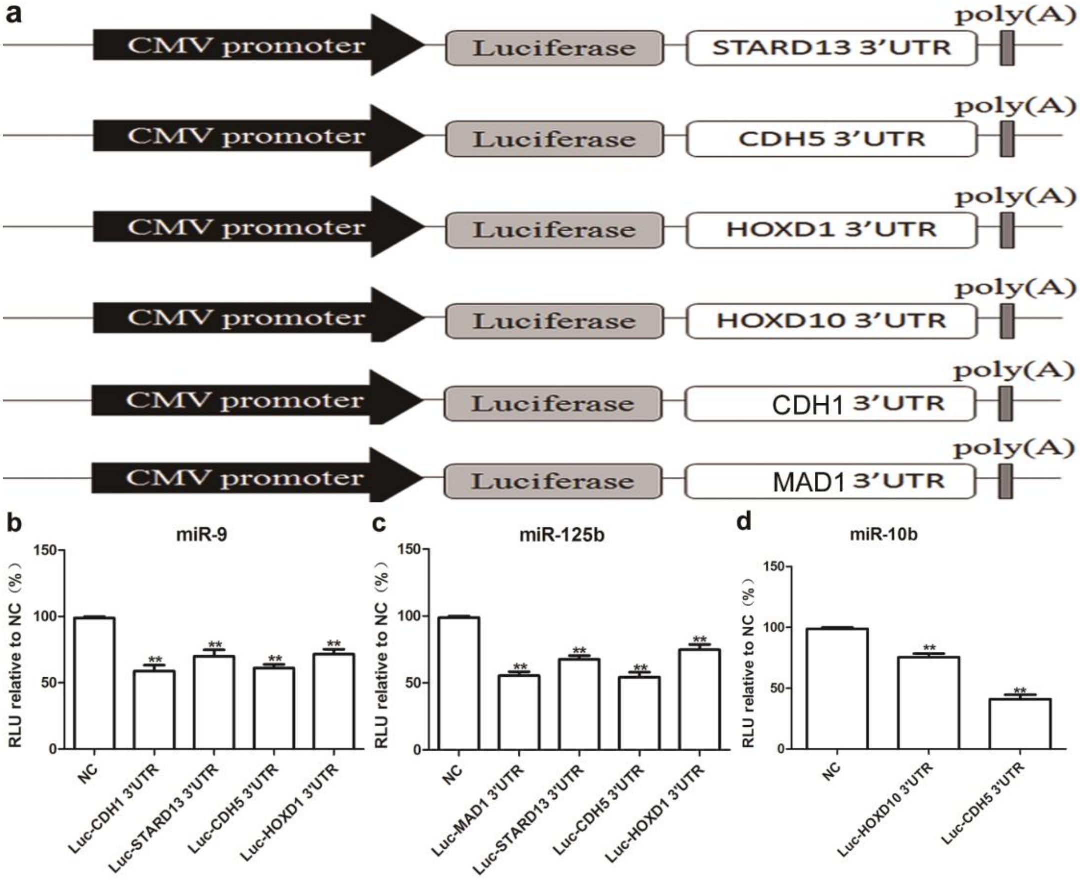

Figure S1: Schematic representation of the luciferase reporter constructs containing fragments of CDH1-, MAD1-, STARD13-, CDH5-, HOXD1-, and HOXD10-3'UTRs which harbored all the predicted miRNAs (a). (b-d) The inhibitory effects of miR-9, miR-10b, and miR-125b on the luciferase activities of CDH1-, MAD1-, STARD13-, CDH5-, HOXD1-, and HOXD10-3′UTRs. HEK293T cells were transfected with the luciferase report constructs of CDH1-, MAD1-, STARD13-, CDH5-, HOXD1-, and HOXD10-3′UTRs along with the corresponding miRNA or miRNA NC. Compared with NC, miRNA could inhibit the luciferase activities of the according targets. n=3, *P < 0.05, **P < 0.01 vs. NC.

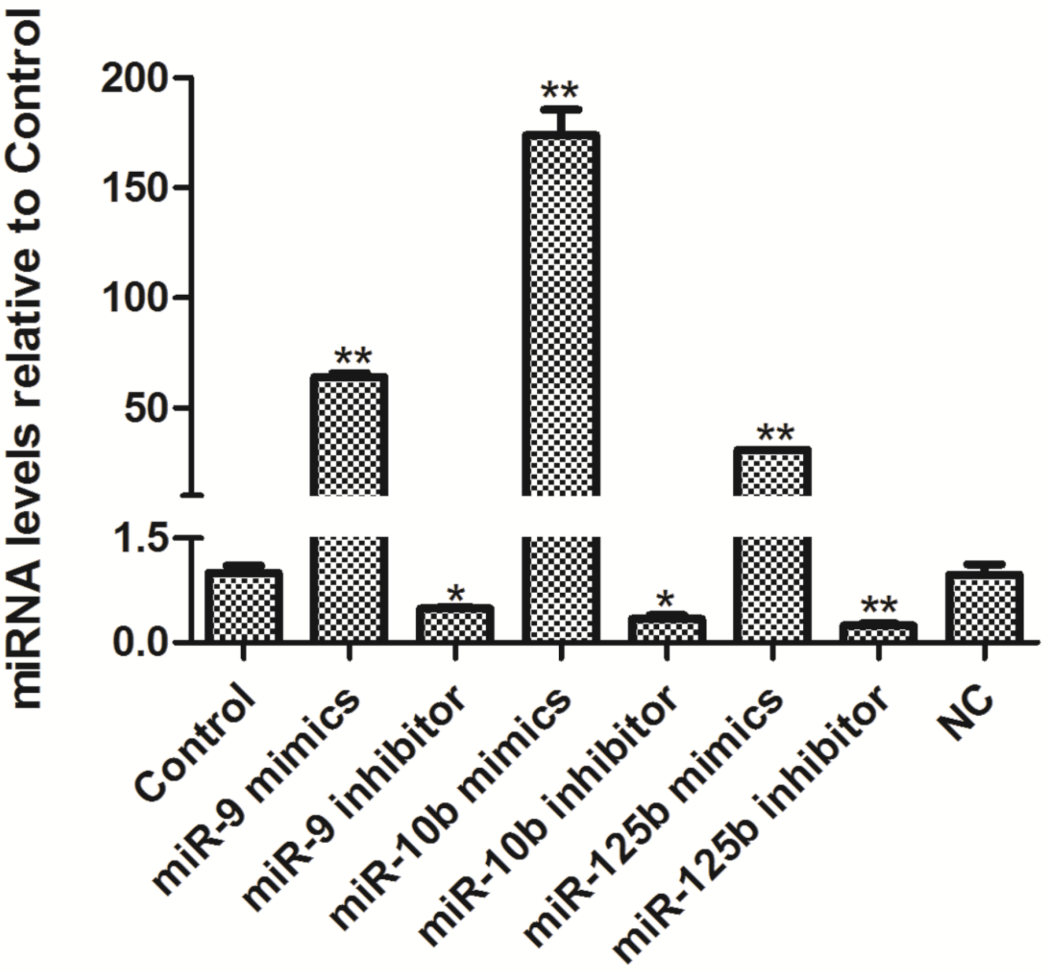

Figure S2: Transfection efficiency of miR-9, miR-10b, and miR-125b. Transfection efficiency of miR-9, miR-10b, and miR-125b was analyzed by miRNA qRT-PCR normalized to U6 in MCF-7 cells. Data were presented as mean ± s.d., ***P* < 0.01 vs. Control.

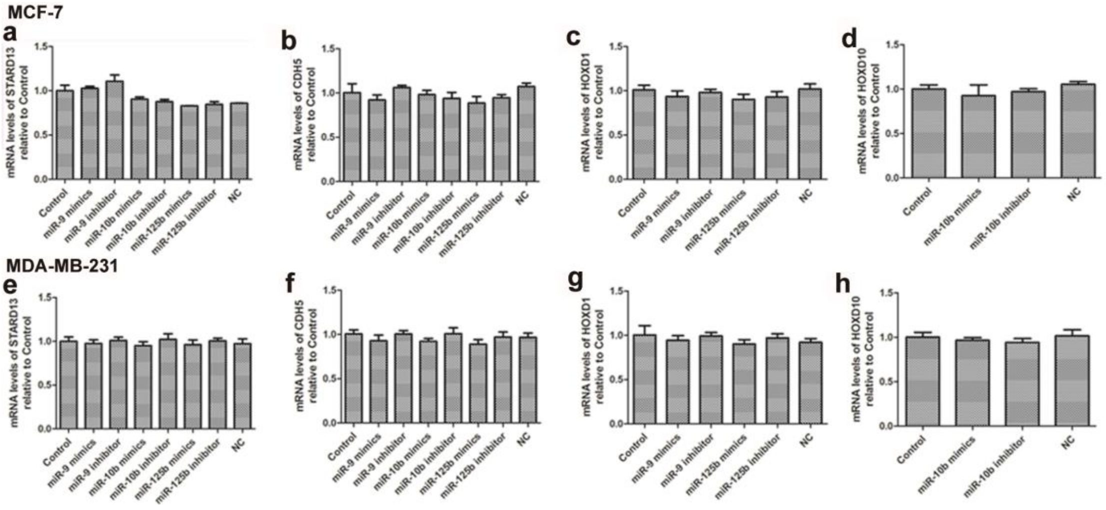

Figure S3: The regulatory effects of miR-9, miR-10b, and miR-125b on the transcriptional levels of STARD13, CDH5, HOXD1, and HOXD10 in both MCF-7 and MDA-MB-231. (a-d) STARD13 (a), CDH5 (b), HOXD1 (c), and HOXD10 (d). (e-h) MDA-MB-231 cells transfected with mimics of miR-9, miR-10b, and miR-125b, inhibitors of miR-9, miR-10b, and miR-125b, and miRNA NC, were subjected to qRT-PCR assay to detect the expression of STARD13 (e), CDH5 (f), HOXD1 (g), and HOXD10 (h).

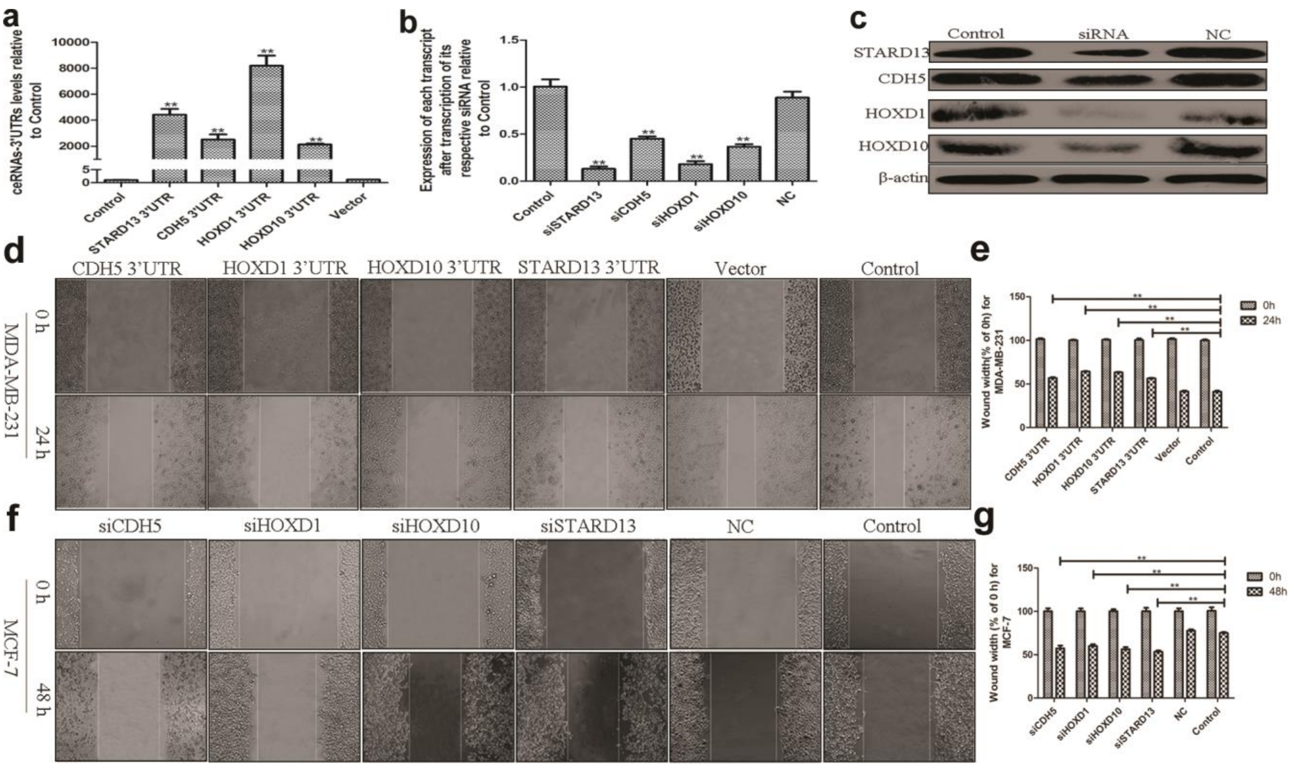

Figure S4: Effects of STARD13 and its ceRNAs on cell migration were analyzed by wound healing assay in both MDA-MB-231 and MCF-7. (a-c) MCF-7 cells transfected with STARD13-, CDH5-, HOXD1-, and HOXD10-3′UTRs, or with siSTARD13, siCDH5, siHOXD1, and siHOXD10 were subjected to qRT-PCR and western blot analysis to confirm the efficient overexpression (a) and knockdown (b and c) of STARD13- and its ceRNAs-3′UTRs. (d) MDA-MB-231 cells transfected with 3′UTRs of STARD13, CDH5, HOXD1, and HOXD10 displayed decreased movement into the wound relative to 0 h compared with the untransfected control cells. (e) Quantitation of cell migration shown in (a). (f) MCF-7 cells transfected with siSTARD13, siCDH5, siHOXD1, and siHOXD10 displayed fast movement into the wound relative to 0 h compared with the control cells. (g) Quantitation of cell migration shown in (c). (b, d) Data were presented as mean ± s.d.; $^{**}P < 0.01$ vs. Control.

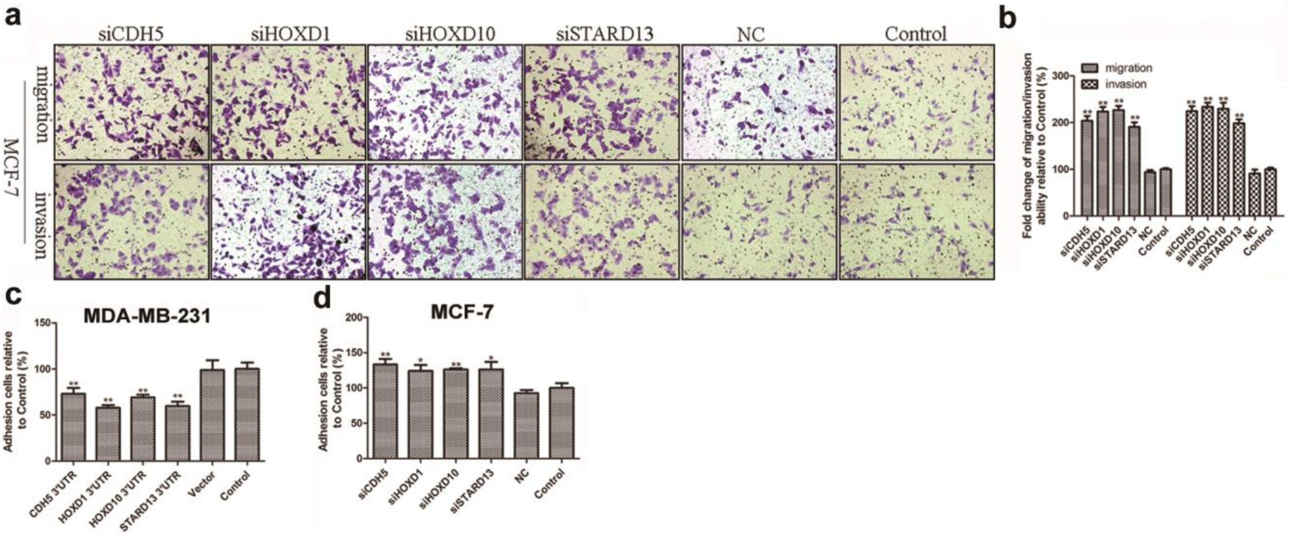

Figure S5: Effects of STARD13 and its ceRNAs on cell migration and invasion were analyzed by transwell assay in MCF-7 cells and cell adhesion was analyzed in both MDA-MB-231 and MCF-7 cells. (a) MCF-7 cells transfected with siSTARD13, siCDH5, siHOXD1, and siHOXD10 displayed an elevated ability of migration and invasion compared to the control cells. (b) Quantitation of cells migration and invasion shown in (b). (c) Ectopic expression of 3′UTRs of STARD13, CDH5, HOXD1, and HOXD10 suppressed adhesion of MDA-MB-231 cells to extra-cellular matrix and basement membranes compared to the control cells. (d) MCF-7 cells transfected with siSTARD13, siCDH5, siHOXD1, and siHOXD10 presented enhanced cell adhesion to extra-cellular matrix and basement membranes compared to the control cells. Data were presented as mean ± s.d., $^{**}P < 0.01$ vs. Control.

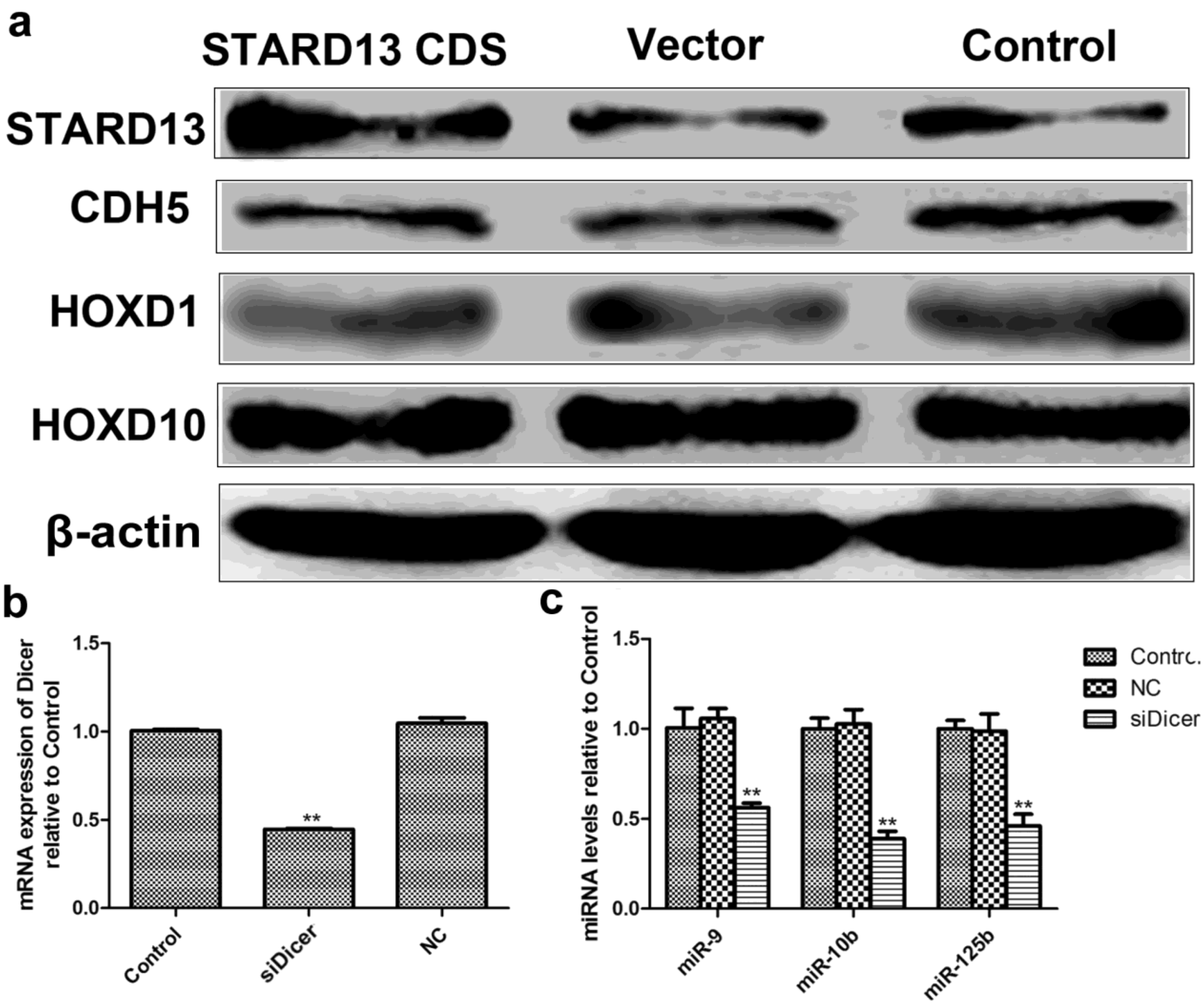

Figure S6: The effects of STARD13-coding region (CDS) on protein levels of CDH5, HOXD1, and HOXD10 and the efficient knowdown of Dicer1 and the concomitant downregulation of miR-9, miR-10b, and miR-125b in MCF-7 cells. (a) Western blot analysis indicated that compared with the untreated control group, ectopic expression of STARD13-coding region (CDS) upregulated protein level of STARD13 without affecting protein levels of CDH5, HOXD1, and HOXD10 in MCF-7 cells. (b and c) MCF-7 cells were transfected with siDicer to block the miRNA biogenesis pathway followed by qRT-PCR for confirmation of Dicer1 knockdown (b) and the concomitant downregulation of miR-9, miR-10b, and miR-125b (c). Data were presented as mean ± s.d., $^{**}P < 0.01$ vs. Control.

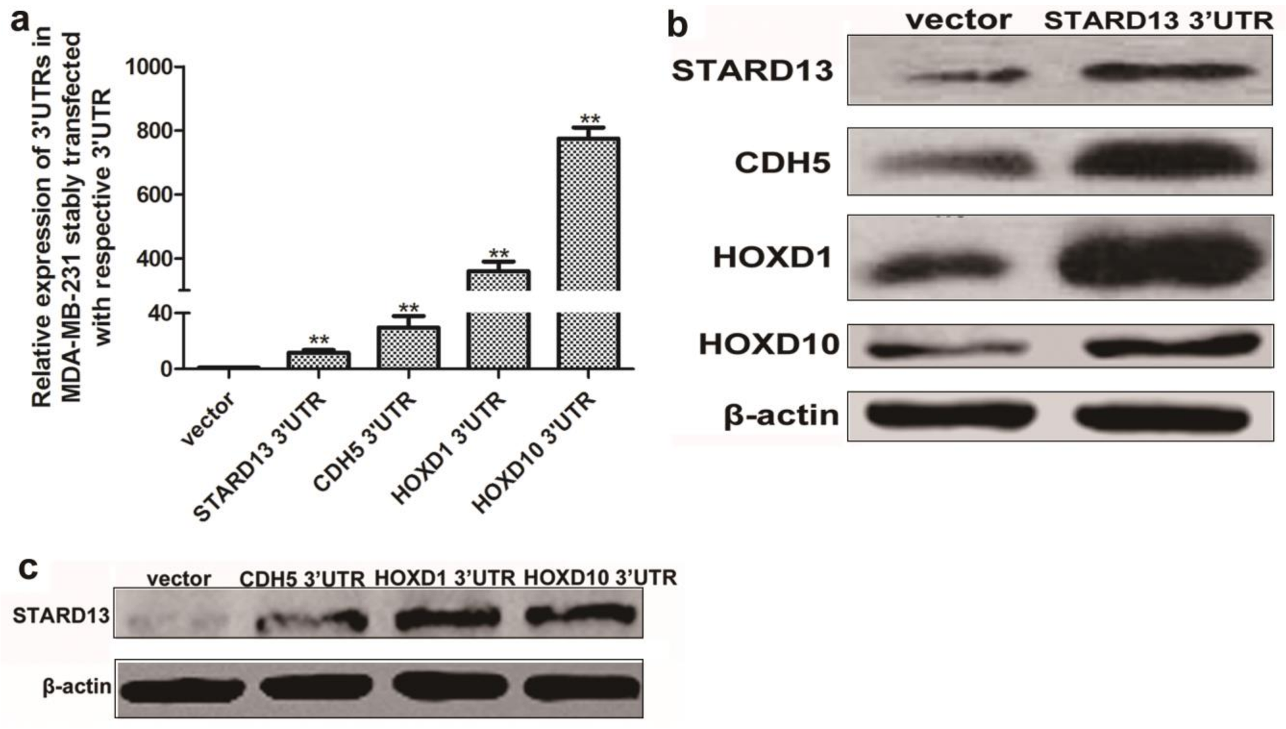

Figure S7: The stable transfection efficiency of STARD13-, CDH5-, HOXD1-, and HOXD10-3′UTRs in MDA-MB-231 cells and reciprocal interaction of ceRNAs *in vivo*. (a) MDA-MB-231 cells stably transfected with STARD13-, CDH5-, HOXD1-, and HOXD10-3′UTRs, or an empty vector were subjected to qRT-PCR analysis to examine the efficient overexpression of STARD13-, CDH5-, HOXD1-, and HOXD10-3′UTRs. Data were presented as mean ± s.d., $^{**}P < 0.01$ vs. vector. (b) Protein levels of STARD13, CDH5, HOXD1, and HOXD10 were analyzed in the tumors transplanted with MDA-MB-231 cells stably overexpressing STARD13 3'UTR. β-actin served as a loading control. (c) Protein levels of STARD13 were analyzed by western blot in the tumors transplanted with MDA-MB-231 cells stably overexpressing CDH5-, HOXD1-, and HOXD10-3′UTRs or empty vector. β-actin served as a loading control.

Supp Video 1. Effects of STARD13-3'UTR on cell migration were monitored by scratch-based migration assays carried out with an Olympus automatic system.

Supp Video 2. Effects of CDH5-3'UTR on cell migration were monitored by scratch-based migration assays carried out with an Olympus automatic system.

Supp Video 3. Effects of HOXD1-3'UTR on cell migration were monitored by scratch-based migration assays carried out with an Olympus automatic system.

Supp Video 4. Effects of HOXD10-3'UTR on cell migration were monitored by scratch-based migration assays carried out with an Olympus automatic system.

Supp Video 5. Effects of Vector on cell migration were monitored by scratch-based migration assays carried out with an Olympus automatic system.

Table S1: Primer sequences used for the construction of the STARD13 ceRNAs-3'UTRs

Gene	Sequences (5' to 3')
STARD13-3'UTR forword	CCCCAGAATGGTACAGCAAAG
STARD13-3'UTR reverse	GAGGGAGAATCAGAAATACAATCAC
CDH5-3'UTR forword	GCTGTACTGAGCACTGAACCAC
CDH5-3'UTR reverse	TCTCTGTTGACTGATGCCACTT
HOXD10-3'UTR forword	GACTTTGGGGTCATTATGTTCG
HOXD10-3'UTR reverse	GGATGCTCTACAGTTCCAATAAGT
HOXD1-3'UTR forword	ACTGTCTTGTAAGCCACTTGTTTG
HOXD1-3'UTR reverse	GCTACATCAAGGAGACCCTAACT
CDH1-3'UTR forword	GGGACTCGAGAGAGGCGGGCC
CDH1-3'UTR reverse	TGAATTGTTTTCCTTTTCCACCCCC
MAD1-3'UTR forword	CCTGCAGGCTCGGGGGCATAG
MAD1-3'UTR reverse	TCTAGGGGAGAAGATTTTATTTCAC

Table S2: Primer sequences used for qRT-PCR

Gene	Sequences (5' to 3')
STARD13 forword	TTAAAGCTAGATGAGGAGTTGCC
STARD13 reverse	TTGACTTGGGGTCTGTAGTGATG
CDH5 forword	CCCATTTTCCAGCAGCCTTT
CDH5 reverse	CGTGTTATCGTGATTATCCGTGA
HOXD10 forword	CCCACTTGCTCCTTCACCAC
HOXD10 reverse	ACGGGCTCGTTCATCTTCTTTT
HOXD1 forword	GTGGATGAAAGTGAAGAGGAATGC
HOXD1 reverse	GAACCAGATTTTGACTTGCGTGT
GAPDH forword	AAGGTCGGAGTCAACGGATT
GAPDH reverse	CTGGAAGATGGTGATGGGATT
